# Proteomic profiling dataset of chemical perturbations in multiple biological backgrounds

**DOI:** 10.1038/s41597-021-01008-4

**Published:** 2021-08-25

**Authors:** Deborah O. Dele-Oni, Karen E. Christianson, Shawn B. Egri, Alvaro Sebastian Vaca Jacome, Katherine C. DeRuff, James Mullahoo, Vagisha Sharma, Desiree Davison, Tak Ko, Michael Bula, Joel Blanchard, Jennie Z. Young, Lev Litichevskiy, Xiaodong Lu, Daniel Lam, Jacob K. Asiedu, Caidin Toder, Adam Officer, Ryan Peckner, Michael J. MacCoss, Li-Huei Tsai, Steven A. Carr, Malvina Papanastasiou, Jacob D. Jaffe

**Affiliations:** 1 Broad Institute of MIT and Harvard, Cambridge, MA 02142 United States; 2 Department of Genome Sciences, University of Washington, Seattle, WA 98195 United States; 3 Picower Institute for Learning and Memory, Massachusetts Institute of Technology, Cambridge, MA 02139 United States; 4 Inzen Therapeutics, Cambridge, MA 02139 United States

**Keywords:** Mass spectrometry, Cancer models, Post-translational modifications, Cell signalling, High-throughput screening

## Abstract

While gene expression profiling has traditionally been the method of choice for large-scale perturbational profiling studies, proteomics has emerged as an effective tool in this context for directly monitoring cellular responses to perturbations. We previously reported a pilot library containing 3400 profiles of multiple perturbations across diverse cellular backgrounds in the reduced-representation phosphoproteome (P100) and chromatin space (Global Chromatin Profiling, GCP). Here, we expand our original dataset to include profiles from a new set of cardiotoxic compounds and from astrocytes, an additional neural cell model, totaling 5300 proteomic signatures. We describe filtering criteria and quality control metrics used to assess and validate the technical quality and reproducibility of our data. To demonstrate the power of the library, we present two case studies where data is queried using the concept of “connectivity” to obtain biological insight. All data presented in this study have been deposited to the ProteomeXchange Consortium with identifiers PXD017458 (P100) and PXD017459 (GCP) and can be queried at https://clue.io/proteomics.

## Abstract (summary)

**Table:** 

Measurement(s)	drug perturbation response
Technology Type(s)	proteomic profiling
Factor Type(s)	cell line • drug
Sample Characteristic - Organism	Homo sapiens

Machine-accessible metadata file describing the reported data: 10.6084/m9.figshare.14744064

## Background & Summary

Dysregulation of post-translational modifications (PTMs), particularly those involved in kinase signaling pathways and epigenetics, is an increasingly common molecular etiology in cancer and neuropsychiatric disorders^1–5^. Protein kinase activity is reflected through phosphorylation, a PTM that can alter protein conformation, subcellular localization, and function, and is implicated in diverse cellular processes including proliferation, differentiation, and death^6^. In chromatin, transcriptional regulation is modulated by PTMs, such as acetylation, methylation, and phosphorylation, on histone proteins that control access of transcriptional machinery to DNA^7^. Highly specific kinase inhibitors and epigenetically-active compounds have demonstrated great therapeutic promise in these areas; for example, tyrosine kinase inhibitors targeting epidermal growth factor receptors (EGFRs) as well as several histone deacetylase inhibitors^8^ have been approved for different cancer therapies^6^. However, these drugs often have off-target effects that can interfere with other normal phosphosignaling and epigenetic activities and are not yet fully understood^6,7,9,10^. Monitoring cellular phosphosignaling cascades and epigenetic modifications in response to drug administration in disease models can therefore illuminate these compounds’ underlying mechanisms of action and predict their efficacies to inform further therapeutic development.

Gene expression profiling has traditionally been used to capture cellular responses to perturbation^11–16^, and while advances in technology have reduced cost and accelerated data generation, a measure of transcription alone cannot fully capture a given cell’s state. Modest correlation between mRNA and protein or phosphorylation levels^17–19^ necessitates complementary readouts. Integrating transcriptomics with proteomics data can fill in the gaps by measuring nucleic acids and proteins in distinct time scales. We previously reported the creation and validation of a pilot library of mass spectrometry (MS)-based proteomic signatures that measure changes in the reduced-representation phosphoproteome (P100)^20^ and changes in epigenetic marks on histones (Global Chromatin Profiling, GCP)^21^ following systematic drug perturbations^22^. In P100, 96 phosphorylated peptides representative of distinct signaling pathways’ activities are measured and provide a reduced-representation of the phosphoproteome in a given cell. In GCP, 79 well-studied combinatorial PTMs (e.g. methylation, acetylation, phosphorylation) of core nucleosomal histones, whose dysregulation is associated with a wide range of diseases, are measured^21,23–28^. This initial pilot library contained signatures of 90 small molecules including kinase inhibitors, epigenetically-active compounds, and neuroactive drugs in 6 cell models–five cancer cell lines (prostate, lung, breast, melanoma, and pancreatic cancer) and one neurodevelopmental cell line (neural progenitor cells (NPC)). This large-scale dataset of more than 3400 signatures facilitated the application of the Connectivity Map concept^11,29^ to our proteomic dataset and allowed for comparisons within and across cell types, drug mechanisms, and assay types^22^. These signatures were contributed to the NIH Library of Integrated Network-Based Cellular Signatures (LINCS), whose mission is to catalog drug-induced cell responses to gain a more detailed understanding of mechanisms underlying disease^30^. Data generated from different assays and across multiple cell types in response to a broad range of perturbations are made publicly available so as to advance basic research and facilitate the identification of therapeutic targets.

In the current study, we expand our initial pilot library to include P100 and GCP profiles generated upon perturbation of the above described cell models with a new set of cardiotoxic compounds, many of which are approved cancer treatments. We further profile all compounds in our library in an additional neural cell model: astrocytes, which play an active role in brain development and are implicated in neurodegenerative disease^31,32^. In total, we have generated more than 5300 profiles corresponding to 118 small-molecule perturbations in 7 different cell lines. We discuss a description of the expanded library, quality control metrics, and case studies demonstrating how this resource can reveal new biological insights and inform new hypotheses.

## Methods

### Cell culture

Cancer cell lines A375, YAPC, A549, MCF7, and PC3 were cultured and treated as described in detail in our previous study^22^. Briefly, A375, A549, and YAPC cells were cultured in RPMI 1640 medium (Thermo Fisher Scientific), MCF7 cells were cultured in DMEM (Thermo Fisher Scientific), and PC3 cells were cultured in RPMI 1640 medium containing 1 mM sodium pyruvate and 10 mM HEPES (Thermo Fisher Scientific). Cancer cell identity was confirmed with fingerprint technology^33^. NPC and astrocyte lineages were differentiated from H9 human embryonic stem cells (WiCell WA09)^34,35^. NPCs were cultured for nine passages in a 1:1 mixture of N-2 and B-27-containing media supplemented with 1 µm dorsomorphin (Tocris Bioscience) and 10 µm SB 431542 (Tocris Bioscience). Astrocytes were cultured in astrocyte medium (ScienCell, Cat No. 1801). The differentiated state of NPCs and astrocytes was confirmed upon staining with appropriate cytological markers^36^.

### Cell treatment and sample preparation for MS analysis

**Fig. 1:**
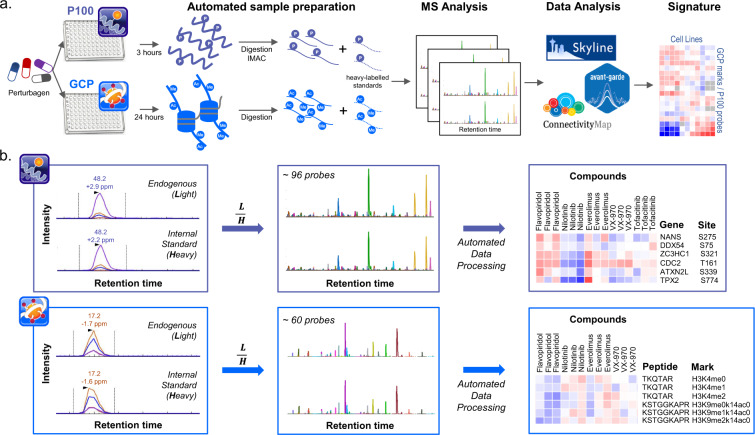
P100 and GCP experimental workflows. (**a**) Processing workflow for P100 and GCP. (**b**) Light (L) and Heavy (H) peptide signal intensities are extracted in Skyline^39^ for individual probes within each sample. Light:Heavy ratios (L/H) calculated in Skyline are filtered using the Proteomics Signature Pipeline (https://github.com/cmap/psp). Processed data are represented in the form of a heat map with each column representing an individual sample and each row an individual probe.

A schematic representation of sample preparation for both P100 and GCP is depicted in Fig. 1a. Cells were plated onto six-well plates for 24 hours, expanded to near confluence, and treated by adding the drug of interest diluted in appropriate media at the desired concentration (Supplementary Table 1). Drugs were selected based on EC_50_/IC_50_ values or effective concentrations used in cellular studies, where known. We further consulted public drug metabolism and pharmacokinetics (DMPK) and absorption, distribution, metabolism, and excretion (ADME) data to select the reported bioavailable concentrations of the drugs in serum. In the absence of prior knowledge, we generally chose 1 μM as a default concentration. Cells were treated either for 3 hours (P100) or 24 hours (GCP). At the end of each treatment period, cells were washed with ice-cold PBS twice for P100 and once for GCP prior to harvest. All treatments occurred in triplicates.

For P100 cell harvest, lysis buffer (8 M Urea, 75 mM NaCl, 50 mM Tris HCl, pH 8.0, 1 mM EDTA, 2 µg/ml aprotinin, 10 µg/ml leupeptin, 1 mM PMSF, 10 mM NaF, Phosphatase Inhibitor Mixture 2 and Phosphatase Inhibitor Mixture 3) was added in each well and cells were collected via scraping. Samples were lysed for 15 minutes at room temperature and then vortexed, followed by an additional 15 minute incubation prior to freezing. Upon thawing, lysates were centrifuged at 15,000 × *g*, 15 °C for 15 minutes to pellet cell debris and extract protein slurry. Protein concentration was measured using the 660 protein assay (Pierce, 22660). All samples (~500 ug each) were normalized to a protein concentration of 1.25 µg/μl. For GCP cell harvest, ice-cold PBS was added in each well and cells were scraped and immediately frozen. Nuclei were extracted following standard protocol^37^. Histones were extracted overnight with 0.4 N H_2_SO_4_ by shaking at room temperature. Solubilized histones were precipitated using 20% w/v trichloroacetic acid for 30 minutes on ice. Samples were centrifuged and the supernatant removed. Histones were air-dried for 10 minutes at room temperature and resuspended in cold HPLC-grade water. Histone protein yield was measured using the Coomassie Plus Protein Assay (Thermo Fisher Scientific).

For P100, proteins were reduced, alkylated and digested overnight with sequencing-grade modified trypsin at an enzyme:substrate ratio of 1:50 (Promega, V511X, Madison, WI). Upon quenching, samples were desalted using reversed phase SPE (Waters, 186002319). Peptides were eluted with 50%ACN/0.1%TFA and lyophilized. Peptides were reconstituted in a quality-control mix of synthetic isotope-labeled peptide standards in 80%ACN/0.1%TFA, used to monitor the recovery of phosphopeptides. Phosphopeptides were enriched using Fe^3+^ IMAC cartridges (AssayMAP Bravo, Agilent, Santa Clara, CA) following standard protocol. Phosphopeptides were desalted using AssayMAP Reverse Phase cartridges (Agilent, G5496-60033) and lyophilized. Prior to MS analysis, a second set of synthetic isotope-labeled peptides were spiked into the samples to allow for quantitation^20^.

For GCP, histones (10 µg) were propionylated by incubating with NHS-propionate at room temperature for 30 minutes. Upon quenching (0.1% TFA), samples were desalted using reversed phase SPE cartridges (Waters, 186000309) following standard protocol. Samples were lyophilized, resuspended in 50 mM ammonium bicarbonate (pH 8.0), and digested overnight with sequencing-grade modified trypsin (Promega) at an enzyme:substrate ratio of 1:50. Peptides were propionylated by incubating with NHS-propionate at 25 °C for 1 hour. Upon quenching (15% hydroxylamine, 25 °C, 30 min), peptides were desalted using SepPak tC18 µElution Plate (Waters) and lyophilized. Prior to MS analysis, peptides were resuspended in a mixture of synthetic isotope-labeled peptides to allow for quantitation. Detailed P100 and GCP protocols can be found online at https://panoramaweb.org/wiki/LINCS/Overview%20Information/page.view?name=sops and in our previous publications^20,21^.

### nanoLC-MS/MS analysis

P100 samples were analyzed on an Orbitrap Q-Exactive HF Plus MS (Thermo Fisher Scientific) and GCP samples on an Orbitrap Q-Exactive Plus (Thermo Fisher Scientific). Both systems were equipped with a nanoflow ionization source (James A. Hill Instrument Services, Arlington, MA) and coupled to a nanoflow Proxeon EASY-nLC 1000 UHPLC system (Thermo Fisher Scientific). Acquisition occurred in positive ion mode with the electrospray voltage set at 2 kV for P100 and 2.2 kV for GCP. Samples were injected onto an in-house packed 20 cm × 75 μm diameter C18 silica picofrit capillary column (1.9-μm ReproSil-Pur C18-AQ beads, Dr. Maisch GmbH, r119.aq; Picofrit 10-μm tip opening, New Objective, PF360-75-10-N-5), heated at 50 °C. The mobile phase flow rate was 250 nL/min for P100 and 200 nL/min for GCP and consisted of 3% ACN/0.1% FA (solvent A) and 90% ACN/0.1% FA (solvent B). Phosphopeptides were separated using the following LC gradient: 0–3% B in 3 min, 5–40% B in 50 min, 40–90% B in 1 min, stay at 90% B for 5.5 min, and 90–50% B in 30 s. Data were acquired using a DIA method to allow for deeper exploration of the phosphoproteome^38^. For MS1 scans, the resolution was set at 60,000 at 200 m/z and the automatic gain control (AGC) target was 3e6 with a maximum inject fill time of 20 ms. An overlap DIA method was used with 56 × 22 m/z isolation windows covering the 400–1,000 m/z range; the isolation windows in two consecutive cycles had an offset of 11 m/z^38^. The default charge state was 4, the resolution was 30,000 at 200 m/z, the AGC target was 1e6, the maximum inject fill time was 50 ms, the loop count was 27, and the NCE was set to 27.

In GCP, histone peptides were separated using the following LC gradient: 3–40% B in 45 min, 40–90% B in 5 min, stay at 90% B for 5 min, and 50% B for 5 minutes. Samples were acquired using a PRM method. Scheduling for each analyte was performed using an inclusion list entailing a mixture of heavy-isotope labeled peptides, with a 60 min window for each analyte. For scheduled samples, the windows were reduced to 3 min for sharp peaks and 20 min for early eluted, wide peaks. A full-scan MS was acquired in profile mode with a resolution of 35,000 at 200 m/z from 280 to 950 m/z, AGC target 1e6, and maximum inject fill time 250 ms. MS2 scans were acquired in centroid mode using a default charge state of 2, resolution 17,500 at 200 m/z, AGC target 1e6, maximum inject fill time 60 ms, loop count 17, and NCE set to 30.

### Data processing

MS raw data files (Level 0 data) were imported into Skyline^39^, and MS2 signals of light and heavy peptides were extracted. The transition refinement and peak integration were performed using Avant-Garde^40^, an automated data curation R package for transition refinement and peak picking for chromatogram-based MS data (Level 1 data). Skyline documents were then imported into PanoramaWeb (https://panoramaweb.org)^41^ for automated downstream processing. For each analyte, the log_2_ ratio of the light to heavy peptide ion signal was calculated, and values for all analytes, along with corresponding metadata, were assembled into Gene Cluster Text (GCT) files (Level 2 data; Fig. 1b). For each batch of samples (per 96-well plate), a single GCT file was generated.

Further filtering and normalization were executed by the python-based Proteomics Signature Pipeline (PSP, available at https://github.com/cmap/psp), integrated into the PanoramaWeb server. Filtering occurred both at the sample and the probe level. Samples with a lower number of probes (<80% for P100 and <50% for GCP) were filtered out, and probes measured in <90% of samples in P100 and <50% of samples in GCP were also discarded. To account for differences in histone loading amounts, samples were further normalized to an invariant peptide (H3, 41-49 and H4, 68-78) in GCP (Level 3 data). All samples were subsequently normalized to the row median value within each plate (Level 4 data). Connectivity scores indicating how similar two perturbations are to each other were subsequently calculated (Level 5 data). For a detailed description of data levels and the connectivity concept, see Litichevskiy *et al*.^22^.

## Data Records

All MS raw files, Skyline documents, and processed GCT files (Levels 0-4 data) are publicly available on Panorama Public^42^ for P100^43^ and GCP^44^ (Supplementary Table 2). These data were deposited to the ProteomeXchange Consortium via Panorama Public^45^ with identifiers PXD017458 (P100) and PXD017459 (GCP). The data can also be found in the LINCS Data Portal with identifiers LDS-41234 (“P100 aggregated data - Chemical perturbations”) and LDS-41235 (“GCP aggregated data - Chemical perturbations”). Connectivity scores (Level 5 data) can be explored and queried using Touchstone-P, part of the Proteomics Connectivity Hub, available at https://clue.io/proteomics.

## Technical Validation

**Fig. 2:**
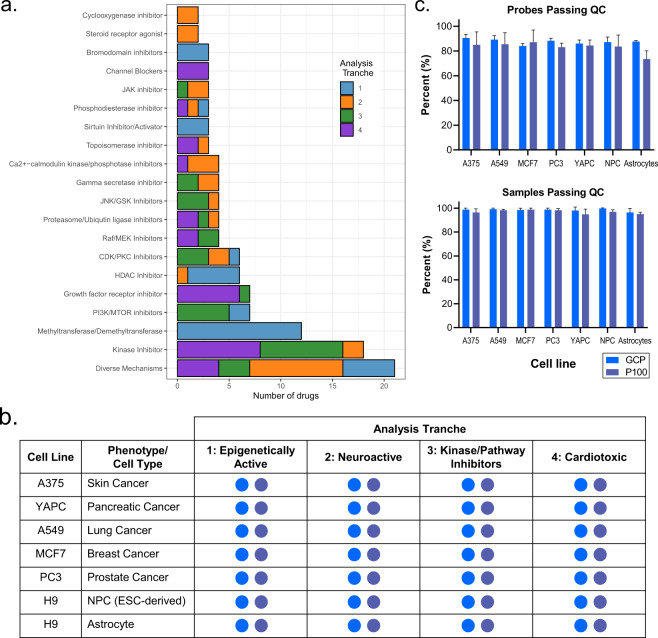
Content and quality control filtering of the phosphosignaling and epigenetics proteomics data library. (**a**) Overview of all mechanisms of action (MOAs) of the compounds employed to build the library. These span four broad categories (epigenetically active, neuroactive, kinase/pathway inhibitors and cardiotoxic), each representing an ‘analysis tranche’ of drugs. The “Diverse Mechanisms” category encompasses MOAs that appear only once in the dataset. (**b**) Overview of the cell lines and drug treatments employed to build the library. Each cell line was treated with all four analysis tranches (29 compounds in each and controls) in 96-well plate batches. Blue circles indicate successful sample processing, acquisition and data analysis for GCP, and purple circles for P100. (**c**) Mean number of probes (assay analytes) and samples (perturbation conditions) passing QC thresholds for each cell type. Error bars represent the standard deviation calculated within each cell type.

Our initial pilot library contained 3400 proteomic perturbational signatures of 90 compounds in six cell lines, including five cancer models (prostate, lung, breast, melanoma, and pancreatic cancer) and one neurodevelopmental model (NPC)^22^. Here, we have expanded our initial library to now include more than 5300 samples, corresponding to profiles generated using 119 small compounds and seven cell lines. The selected compounds encompass diverse mechanisms of action (MOAs), but common groups of MOAs emerge that represent mechanisms directly modulating epigenetic processes (e.g. HDAC inhibitors and methyltransferases/demethyltransferases) and phosphosignaling pathways (e.g. JAK and Raf/MEK inhibitors) (Fig. 2a). In the expanded dataset, we profiled a new set of 29 cardiotoxic compounds, many of which are approved chemotherapeutics^46^, with the intention that our data can support ongoing pharmacology efforts aiming to develop novel, non-toxic therapeutics^47^. All compounds were further profiled in a second neural cell model, astrocytes, which are a major cell type of the central nervous system whose dysregulation is implicated in neurodegeneration and other pathologies^31^. Although astrocytic proteomes from healthy and disease models derived from different biological sources have been monitored^36,48,49^, to the best of our knowledge, epigenetic and phosphoproteome changes elicited upon drug perturbations of such large extent have not been reported yet. This dataset complements profiles obtained from NPCs using the same set of perturbations and allows for a direct comparison of neural lineage differentiation.

Samples were processed in batches of 96-well plates, with each plate corresponding to one set of compounds, referred to as an “analysis tranche,” profiled in one cell line (Fig. 2b). Using strict criteria, we initially filtered our data based on the number of samples in which a probe was detected in each plate and the number of probes detected within a sample (Data Processing, Methods). On average, 83% and 88% of the probes passed our filtering thresholds in P100 and GCP respectively, indicating high quality data (Fig. 2c). The lower percentage of probes passing QC in P100 compared to GCP can be explained by the more stringent threshold employed (80% in P100 vs. 50% in GCP). Interestingly, fewer P100 probes passed the filtering threshold in astrocytes compared to their progenitor cells (NPCs), showing particular sensitivity to epigenetic compound perturbations. Epigenetic compounds, such as HDAC and methyltransferase inhibitors, did not induce strong changes to the P100 phosphosignaling landscape, resulting in noisy signatures with poor reproducibility within probes. In contrast, a comparable number of probes passed filtering criteria in NPCs and astrocytes in GCP, suggesting a more conserved epigenetic landscape across cell lines, as compared to the cell type-specific reduced phosphoproteome. Overall, more than 95% of the samples across all cell lines passed filtering thresholds, yielding near-complete datasets with few sample failures.

**Fig. 3:**
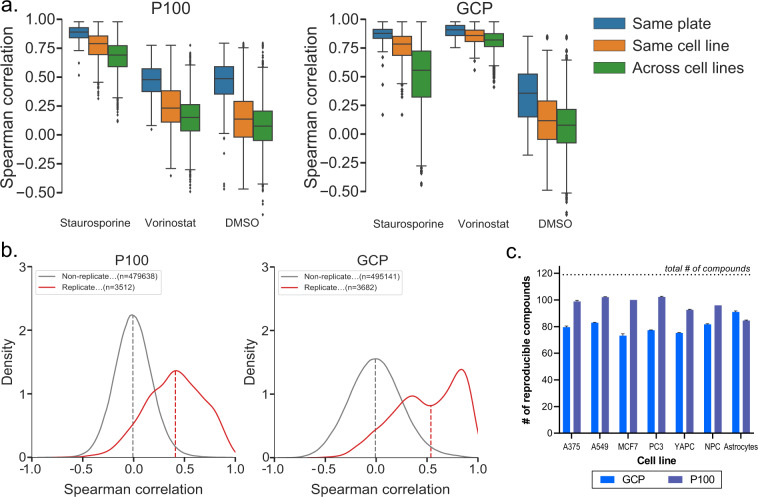
Quality assessment of the LINCS signaling and epigenetics proteomics data library. (**a**) Correlation of replicates for experimental controls employed in the library. Boxplots show the distribution of Spearman correlation coefficients for replicates within the same plate, within the same cell line, and across all cell lines. Boxes indicate the extents of the 1st and 3rd quartile, while whiskers indicate 1.5x the interquartile range. (**b**) Distributions of all Spearman correlations among replicates (red) and among non-replicates (gray) across the whole dataset, with dashed lines representing the median of the distribution. (**c**) Bar chart showing the number of compounds considered reproducible in each cell line for each assay. The permutation test was run 10 times with 10,000 bootstrapped iterations; bars represent the average and error bars represent the standard deviation of the 10 runs.

Within each plate, we included a DMSO vehicle control to obtain baseline measurements relative to which perturbation profiles could be compared. To assess technical reproducibility over the course of data generation and enable comparisons between samples, we also included two positive controls within each plate, one specific to each assay. The P100 positive control was staurosporine, a molecule that inhibits a variety of kinases inducing widespread phosphoproteome changes^50^. For GCP, we used vorinostat, which inhibits the enzymatic activity of histone deacetylases (HDACs)^51^, which catalyze the removal of acetyl groups from lysine residues. The controls allowed for comparisons to be made within a plate (same cell line, one set of drugs), within a cell line (same cell line, all sets of drugs), and across all plates (all cell lines, all sets of drugs) (Fig. 3a).

Staurosporine profiles were highly reproducible in P100 (calculated using Spearman correlation), with higher values calculated within a plate (0.89) and cell line (0.79) and a slightly lower value (0.69) across different cell lines, which is expected due to varying genetic cellular backgrounds (Fig. 3a). Despite its role as a P100 control, staurosporine also had a particularly reproducible signature in GCP within a plate (0.88) and cell line (0.78). Long treatment periods with staurosporine (24 hours) have been implicated in cell death^20^, and we observe a characteristic epigenetic profile of reduced phosphorylation on H3 S10, a marker of proliferation^52^. Staurosporine causes direct inhibition of aurora B kinase which is responsible for S10 deposition, a factor contributing to cell death^53^. Lower reproducibility (0.57) was observed across all cell lines in GCP, indicating that different cells may have different epigenetic responses to staurosporine. In contrast, vorinostat was highly reproducible in GCP overall (>0.82), with tight distributions at all three levels, due to a strong increase in acetyl marks upon HDAC inhibition. Vorinostat correlated poorly in P100, as expected, since acetyl states are not monitored in this phosphoproteomics assay. Finally, DMSO, which represents the baseline profile of a cell line, correlated poorly at all three levels due to its “null” signature that reflects little change in the cells.

The technical quality of our expanded dataset was then assessed by comparing the distributions generated by replicate and non-replicate correlations (Spearman). In both assays, distributions were well separated with median values of replicates at 0.41 for P100 and 0.54 for GCP, and non-replicates at 0 (Fig. 3b). In GCP, we observed a bimodal distribution, with two local maxima detected at 0.39 and 0.84 indicating two groups of compounds. The left mode originated from compounds that induce relatively minor changes to histone marks (e.g kinase inhibitors), as they target specific pathways that are not expected to impact epigenetics. The right mode comprised mainly HDAC inhibitors and other epigenetically active compounds, which induce predictable and strong signatures in GCP. Overall, the distributions calculated here resembled our pilot library^22^, suggesting that the addition of cardiovascular drugs and astrocytes did not affect the overall distribution of the library.

We further tested reproducibility by performing 10 random permutation tests of all samples compared with the permutation null and investigated whether true replicate correlations were at the highest 5% of a distribution (q value < 0.05). If so, the compound was considered “reproducible”. Control samples contributed 12 replicates per cell line, while compounds contributed 3 replicates per cell line. We observed > 70% reproducibility in all cell lines, with fewer compounds reproducible in GCP due to compound classes (e.g. kinase inhibitors) that did not induce large changes in the chromatin space (Fig. 3c). Overall, the quality metrics described here revealed reproducible signatures within and across plates, increasing confidence in the quality of data produced.

## Usage Notes

In the current study, we expanded our pilot library of P100 and GCP data, originally consisting of 3400 samples, to a total of 5300 samples. Levels 0-4 data are made available as a resource to the research community on Panorama Public (see Data Records). GCTs of filtered and normalized data (Level 4) can be downloaded from Panorama Public and visualized as a heatmap in Morpheus, a software tool developed at the Broad Institute and accessible at https://clue.io/morpheus. Data visualization allows us to easily identify how specific phosphosites or epigenetic markers respond to unique perturbations. In addition to visualization, Morpheus also offers various data analysis options such as hierarchical clustering and marker selection.

Level 4 data can be queried using the Touchstone-P query tool at https://clue.io/proteomics to explore connectivity between drug signatures. The query returns connectivity values that researchers can use to identify drugs with signatures strongly connected or anti-connected to their compound of interest, as well as noting if the top hits have similar or different mechanisms of action; examples are given in the case studies below. Overall, this study provides an extensive proteomics library cataloging cellular responses to compounds involved in treating cancer and other diseases. We anticipate that this library will be used to confirm biological mechanisms and also raise new hypotheses for further investigation.

### Use case 1: GCP query of external data

**Fig. 4:**
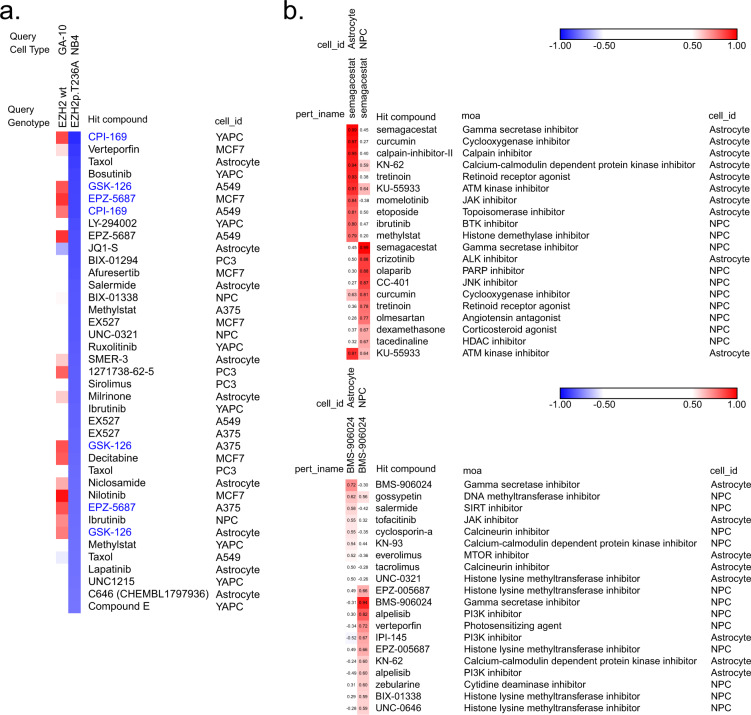
Use case illustrations for GCP and P100 data query. (**a**) Connectivity query of chromatin signatures of EZH2 wild-type (GA-10) and EZH2 mutant (NB4) cell lines from the Cancer Cell Line Encyclopedia (CCLE)^54^. This query illustrates how the library can be used to validate a presumptive gain-of-function mutation. Results are sorted from bottom to top ranks for the NB4 line (bottom 5% shown here) and identify EZH2 inhibitors (CPI-169, EPZ-005687, and GSK-126, highlighted in blue) as the most anti-connected hits. (**b**) Query results and connectivity matrix of two gamma secretase inhibitors, BMS-906024 and Semagacestat, in NPCs and astrocytes. For both drugs, the first ten rows correspond to the top ten most connected drugs to astrocytes, and the bottom ten rows to the top ten most connected drugs to NPCs. This query illustrates how the library can provide insight to a compound’s mechanism of action in differentiated cell types.

The Touchstone-P query tool can be employed by investigators to classify the epigenetic signatures of their own samples, as GCP and GCP-like data is relatively common in the chromatin proteomics field. For example, over 800 cell lines have been profiled as part of the Cancer Cell Line Encyclopedia (CCLE) project^54^. In Fig. 4a,Touchstone-P is used to evaluate whether the CCLE cell line NB4, harboring an EZH2 mutation (T236A), acts via a gain-of-function mechanism, in which case EZH2 activity should be increased. To investigate this, we performed a query of the wild-type EZH2 cell line, GA-10, and the mutant EZH2 line, NB4, against our LINCS database and obtained connectivity values for each compound in the library. Negative connectivity values of NB4 with EZH2 inhibitors, compounds that have been shown to decrease the activity of the wild type enzyme^55^, would point to a gain-of-function mechanism for NB4^55^. Indeed, sorting NB4 connectivity values in ascending order, four of the top ten hits are identified as EZH2 inhibitors (CPI-169, EPZ-005687, GSK-126) showcasing the power of Touchstone-P for deciphering the functional implications of mutations. If no genetic information was known about the NB4 line *a priori*, the EZH2 gain-of-function mutation could have been predicted from these results. Similarly, researchers can use this tool to establish predictions about their cell system when no other classifications are available.

### Use case 2: Exploring cell type-specific responses using P100

NPCs give rise to many of the major cell types in the central nervous system, including astrocytes^56^, which support both neuronal signaling and cerebrovascular integrity^57^. Due to the difficulty in obtaining primary human astrocytes, *in vitro* differentiation of NPCs into astrocytes is preferred for investigation. To date, no proteomics studies that compare isogenic NPCs and astrocytes in response to drug perturbations have been reported. We were interested in leveraging the LINCS library to compare signaling patterns between these two cell types upon perturbation by drugs with similar mechanisms of action (MOAs). We focused our analysis on two gamma secretase inhibitors given the gamma secretase pathway’s relevance in neural differentiation^58^, and we queried the profiles of NPCs and astrocytes treated with BMS-906024 and semagacestat against the entire phosphoproteomic library. Replicate profiles were highly reproducible, as shown by high connectivity values of each drug to itself within each cell type; however, across cell types, lower connectivity values were observed for each compound, with semagacestat demonstrating low positive connectivity to itself between cell types and BMS-906024 showing negative connectivity (Fig. 4b). Differences in the morphology and biological function of NPCs and astrocytes could explain their differing cellular responses to the same drug. For example, in NPCs gamma secretase inhibitors can suppress NOTCH-1 signaling to support differentiation^59^, while in astrocytes these inhibitors can prevent secretion of amyloid-beta^60^. Moreover, the differences in connectivity patterns displayed by semagacestat and BMS-906024 between cell types could suggest that gamma secretase inhibitors have unique selectivities that modulate their activities in different cell types. Since it is known that the gamma secretase complex is composed of many components that can regulate each other, with different isoforms leading to alternative function^61,62^, perhaps semagacestat acts more universally in inhibiting the gamma secretase complex to produce more similar signatures in different cell types than BMS-906024 does.

Several unexpected connections were also observed for both drugs in both cell types, which could provide insight into secondary MOAs implicated in potential off-target effects. In both NPCs and astrocytes, BMS-906024 showed strong connections to several histone lysine methyltransferase inhibitors, and semagacestat showed off-target connectivity to a number of different phosphosignaling pathway inhibitors (Fig. 4b). Unintended responses to a drug can have harmful clinical implications; for example, semagacestat failed a clinical trial for treatment of Alzheimer’s disease because it not only failed to slow disease progression, but also demonstrated an increase in adverse events such as development of skin cancers and infections^63^. Our library can thus be a useful resource to reveal potential off-targets for further investigation and to provide insight into underlying MOAs that could contribute to side effects of drug administration.

This example query of gamma secretase inhibitors in neural cell types demonstrates how our expanded library can be used to reveal differing responses across different cell types elicited by compounds with the same MOA. It is emerging that neurological diseases often impact several different cell types in the human brain. Understanding how each cell type responds to pharmacological perturbations and whether a drug exhibits a synchronous (connected) or dyssynchronous (anti-connected) response across multiple cell types will be critical for developing the next generation of therapeutics for neurological diseases.

## Supplementary information

Supplementary Table 1

Supplementary Table 2

## Data Availability

The Proteomics Signature Pipeline (PSP) is available online at https://github.com/cmap/psp. *Avant-garde* is available at https://github.com/SebVaca/Avant_garde and can be downloaded from the Skyline Tool Store directly in the Skyline interface or at https://skyline.ms/skyts/home/software/Skyline/tools/details.view?name=AvantGardeDIA.
